# A protocol for a randomized clinical trial of interactive video dance: potential for effects on cognitive function

**DOI:** 10.1186/1471-2318-12-23

**Published:** 2012-06-06

**Authors:** Jelena Jovancevic, Caterina Rosano, Subashan Perera, Kirk I Erickson, Stephanie Studenski

**Affiliations:** 1 School of Medicine, University of Pittsburgh, Pittsburgh, USA; 2 School of Public Health, University of Pittsburgh, Pittsburgh, USA; 3 Department of Psychology, University of Pittsburgh, Pittsburgh, USA

## Abstract

**Background:**

Physical exercise has the potential to affect cognitive function, but most evidence to date focuses on cognitive effects of fitness training. Cognitive exercise also may influence cognitive function, but many cognitive training paradigms have failed to provide carry-over to daily cognitive function. Video games provide a broader, more contextual approach to cognitive training that may induce cognitive gains and have carry over to daily function. Most video games do not involve physical exercise, but some novel forms of interactive video games combine physical activity and cognitive challenge.

**Methods/Design:**

This paper describes a randomized clinical trial in 168 postmenopausal sedentary overweight women that compares an interactive video dance game with brisk walking and delayed entry controls. The primary endpoint is adherence to activity at six months. Additional endpoints include aspects of physical and mental health. We focus this report primarily on the rationale and plans for assessment of multiple cognitive functions.

**Discussion:**

This randomized clinical trial may provide new information about the cognitive effects of interactive videodance. It is also the first trial to examine physical and cognitive effects in older women. Interactive video games may offer novel strategies to promote physical activity and health across the life span.

The study is IRB approved and the number is: PRO08080012

ClinicalTrials.gov Identifier: NCT01443455

## Background

Physical exercise has the potential to affect cognitive function, but most evidence to date focuses on cognitive effects of aerobic training. [1-3] Cognitive exercise also may influence cognitive function, but many cognitive training paradigms have failed to provide transferto daily cognitive function or have small effects. [2-4] Video games provide a broader, more contextual approach to cognitive training that may induce cognitive gains and have carry over to daily function. [3,5] Most video games do not involve physical exercise, but some novel forms of interactive video games combine physical activity and cognitive challenge [5,6].

The purpose of this report is to provide an overview of a randomized clinical trial in postmenopausal sedentary overweight women for effects on physical and cognitive function, as well as other outcomes, of an interactive video dance game compared to brisk walking and delayed entry controls. We focus primarily on the rationale and plans for assessment of multiple cognitive functions.

### Potential cognitive effects of videodance games

Interventions to increase cognitive and sensorimotor skills are of interest because they may be of benefit to persons with neurological damage or to slow or reverse age-related declines in visual, cognitive or motor skills. Skill learning is defined as an improvement in perceptual, cognitive or motor performance as a result of training [7]. The effects of training persist for several weeks or months, as opposed to adaptation, which is maintained only for brief periods [8]. Novel skills are learned more quickly when trained under variable conditions and variable priority training leads to greater transfer and longer retention [9]. Learning should generalize to other tasks that are based on similar relationships among cognitive, sensory and motor systems. Overlap among these cognitive processes is the most important aspect of training and transfer. See the excellent review by Lustig et al [10]. However, generalization does not occur with some types of training sessions, resulting in significant barriers to the design of learning paradigms. [11-13] Most often, for generalization to occur, sensorimotor training tasks should include system interactions that resemble aspects of the target general tasks.

However, in order to design a successful learning paradigm, several obstacles must be overcome [7]. First, despite humans' exceptional capacity for learning, this learning tends to be highly specific. In the field of perceptual, motor and cognitive learning, various studies have found no or minimal generalization across conditions. Further, training tasks can be boring, and unpleasant, decreasing the compliance with the regimen, leading to poor results. Thirdly, other factors such as mood and motivation can influence performance. When designing a training paradigm, it is important to consider these issues.

Some learning paradigms promote generalization to novel tasks. Such paradigms tend to be more complex and variable than many structured laboratory training tasks, and often relate to real world experiences [4,7]. Several forms of complex training that appear to demonstrate generalization and carry over include action video games, music and sports [7,14].

### Visuomotor integration

Visual information is important for navigating toward and around objects in the environment. The visual system must deal with natural, complex scenes in everyday life, where one is surrounded by a variety of potentially relevant stimuli. How does the visual system monitor the environment and guide behavior, given constraints within attentional and working memory systems? The visual system must detect information about the features of an object and provide appropriate feedback for the control and guidance of movement at the time when it is needed. The motor system uses that information to coordinate the appropriate movement to maneuver. This issue is particularly relevant to locomotion and fall prevention. Observers learn to represent sufficient structure about the visual environment in order to guide eye movement in a pro-active manner, in order to fixate critical regions at the right time [15]. The accuracy of perceptual-motor coordination depends on how well the person updates a changing distance from the object [15]. Adaptation of the perceptual-motor systems occurs when biomechanical activities adjust to changing visual information in the environment. By utilizing the information from the environment, a person perceives changes in body position to target location and thus can avoid obstacles. Previous studies have examined practice strategies to improve visuo-motor learning of novel tasks/skills [16,17].

### Video games as a tool to train cognitive skills

Not all video games equally influence cognitive skills; learning depends on the goals of the game with effects on different cognitive functions (Table 1).

**Table 1:** Evidence about the effects of playing action video games on aspects of cognition

**Marked improvement**	**No or little effect**
Attention [18-21] (divided and selective attention)	Judgment and problem solving skills [22]
Visuo- spatial skills (speed of processing [19,23-25], reaction times [20,26], mental rotations [22], processing in the periphery [19,21])	Exogenous attention [25,26] (alerting and orienting)
Short term memory [27,28]	

Cognitive effects can be assigned to one or more of five general categories [29]. First, action video games require the player to monitor and react to multiple objects in the periphery, and thus have the highest impact on visual and attentional systems. Second are sports and racing video games, which have the highest impact on visual processing and reaction times. Third are games like Tetris that require rapid visuo-motor processing. They affect capacity for mental rotation and memory, but are not as attentionally demanding as action videos games, due to the limited number of objects and actions. Fourth are strategy games, with the highest impact on cognitive tactics and planning. Fifth, puzzle games promote problem solving strategies, assisted by mental imagery.

Interactive videodance games are a form of action video game that also require physical activity. These games require constant monitoring of the periphery for frequent unpredictable events that require quick and accurate responses. This monitoring places heavy demands on visuo-attentional systems, as players need to keep track of many moving objects while ignoring distracters. These games also require precise visuo-motor control in order to aim steps in space and time according to the sequence of moving targets. There is a growing interest in the role of action video game playing in training perceptual and cognitive abilities [30]. Prior observational studies suggest that video games may affect a wide variety of different skills including improved hand- eye coordination [31], increased processing in the periphery [18], enhanced mental rotation skills [22], greater divided attention abilities [19,20] and faster reaction times [23,24,26].

Action video games also appear to enhance attention in a variety of tasks. For example, action game players outperform their peers on a multiple-object tracking task, where participants must track many independently moving objects [27]. Further, as a result of faster reaction times, they process distractors flanking the targets more efficiently [19] . Action video game players have also been shown to perform better on the useful field of view task [18], which indexes a person's ability to deploy attention over space [21]. Video gaming may therefore provide an efficient training regimen to induce a general speeding of perceptual reaction times without decreases in accuracy of performance.

Some video games combine multiple strategies and may affect multiple cognitive domains. For example, play may require simultaneous performance of executive tasks (decision making, dual tasking), visual attention tasks (multiple object tracking, distractor rejection), visuomotor tasks (e.g. navigating), and rapid object recognition. Our clinical trial is designed to examine the effects of video based dancing on several aspects of cognition using a set of cognitive performance measures.

### Neuroimaging Paradigms to Test Effects on Brain Function

Interactive videodance relies on rapid decision-making under divided attention conditions, requires multiple object recognition and tracking, and distractor rejection. Video dance also places demands on aerobic fitness, which may also enhance brain function [1,3]. This clinical trial hypothesizes that the combined cognitive/exercise intervention of interactive video dance will lead to improved visuo- and perceptual-motor speed-of-processing and will be associated with enhanced functional activity in regions important for these domains, specifically the cortical fronto-parietal networks [32,33].

Previously, we performed a functional MRI study ancillary to a pilot uncontrolled trial of interactive video dance using the computerized version [34] of the digit symbol substitution task (DSST), a test of psychomotor performance. We chose this test for several reasons: first, because the DSST test is particularly sensitive to cognitive changes associated with aging [34,35] and it relies on functional domains (incidental memory, perceptual organization, visuomotor coordination, selective attention and ability to filter out irrelevant information) [36] that overlap with those required to perform videodance. This test also has high test-retest reliability [37] and has been validated it in independent studies [38,39]. Specifically, there is a significant correlation (p < 0.001) between the accuracy of the computerized version of this test and the pencil and paper test score. Finally, we have shown that in older adults, greater DSST accuracy is associated with greater fMRI signal within specific information processing regions, including the left dorsolateral prefrontal cortex [40] and that a physical activity intervention [38] is associated with greater DSST performance and neural activation within these areas.

The pilot study obtained brain fMRI data of 5 women (mean age: 55.4 years, std deviation: 6.46 years) before and after vidoedancing for 6-weeks, twice a week (average number of days between scans: 54 days with standard deviation 11.59 days). The task and the Region of interest analyses have been described elsewhere. [38-43] Briefly, fMRI were acquired with a 3 T Siemens Trio MR scanner using EPI sequence (axial view, TR = 2 s, TE = 32 ms, thickness = 3 mm) and a block design task of the computerized version of the DSST. Accuracy (number of corrected symbols) and reaction time (time in seconds to respond) were measured. The data was preprocessed (motion corrected, normalized and smooth) and analyzed using SPM5 [44]. Participants were instructed on the task outside the magnet for as long as needed to familiarize them with the task (usually 5–10 min).

**Figure 1:**
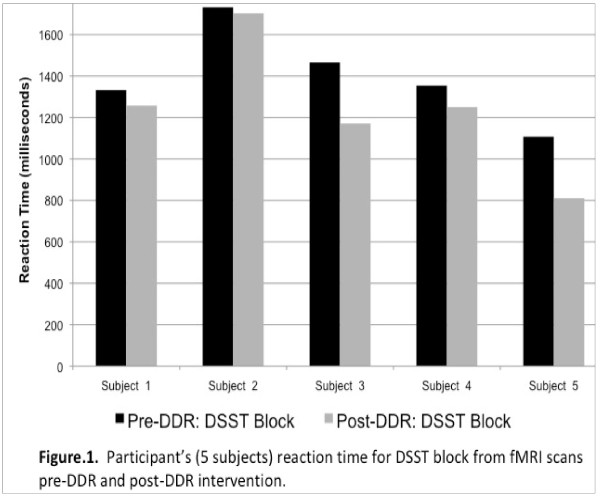
Participant’s reaction time pre and post dance intervention.

**Figure 2:**
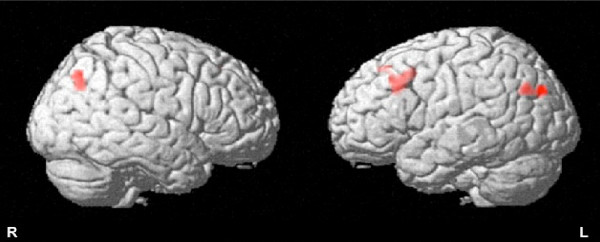
Spatial distribution of functional brain MRI activation.

All 5 participants responded more quickly (Figure.1) and showed greater brain activation (Figure.2) in the post- vs. pre-intervention testing. Differences in activation were significant in the hypothesized regions (left middle frontal gyrus, right inferior parietal lobule) as well as in the left angular gyrus. Accuracy among these midlife women was already 100% (0%) before the intervention and remained high afterwards [98.38% (2.31%)].

Our pilot findings suggest that interactive videodance is associated with increased fronto-parietal network activation and a trend towards improved reaction time. We also learned that this specific task may be too ‘easy’ for midlife women. In the ongoing clinical trial we have modified our approach to add a task-switching component to the fMRI paradigm. Faster performance on task-switching tasks was associated with higher physical activity levels, in one study [45] And expert videogamers outperform their more novice peers on paradigms involving task-switching [28] Given the importance of preparing for and switching tasks in the DDR game, we hypothesize that the task-switch paradigm would test whether the DDR intervention improves cognition, and executive function in particular.

## Methods/design

This is a 6 month randomized clinical trial comparing video dance, brisk walking and delayed entry controls. The interventions have two phases; a 12 week initiation phase with substantial structure and supervision, followed by a 12 week transition phase, with reduced structure and supervision. Participants are 168 overweight or obese, sedentary postmenopausal women aged 50 to 65; 60 in each exercise arm and 48 in the wait list control group.

The following research questions will be assessed:

1. Is exercise adherence at 6 months better with video dance games compared to brisk walking?

2. Does video dance game exercise compared to wait list controls, induce beneficial changes in physical and mental health?

3. Does video dance game exercise compared to brisk walking better promote balance, attention and visual spatial skills, without loss of benefit to cardiovascular fitness?

4. Is video dance preferred to brisk walking for exercise among postmenopausal women? If so, who and why?

### Rationale

Post menopausal women are an important target group for promoting physical and mental health through exercise. The menopausal transition and beyond is associated with adverse changes in body composition (bone, fat and muscle), worsening vascular health and negative effects on glucose and lipid metabolism. [46-48] Adverse symptoms related to cognition, mood and sleep increase in frequency. [49,50] It is well known that spontaneous physical activity decreases with aging in all animal species, including humans, suggesting that there are underlying age-related processes that affect desire for activity. [51] Women are less active as they age; over 30% of women >50 years old report no physical activity in the last month, inactivity continues to increase with further aging and is higher among ethnic minorities than Caucasians. [52,53] Barriers to physical activity abound; women report that exercise can be boring and they don’t like to compete with others. They prefer challenge without competition, fun, opportunities to focus on oneself as well as opportunities to interact with others [54]. Menopausal women report problems with time, motivation, competing obligations, and environmental barriers. [55] Dancing appeals to some women as a form of exercise. Some forms of dance promote fitness, strength, balance, weight loss and possibly bone health in postmenopausal and aging women [56-59]. In our pilot study of videodance in postmenopausal women, over 90% of participants said dancing was fun and that they would recommend dancing to others; 75 + % asked to continue dancing, liked to choose music and dances, felt dancing helped to meet their physical activity goals, and helped improve coordination, attention, fitness and weight. [60] Dancing has been reported to be associated with reduced risk of dementia. [59] Dancing is also remarkably safe, with a low injury rate. [10,61] In addition, the aesthetic pleasures of music and dance may play an important role in motivation. [62]

Interactive video dance games may serve as an attractive exercise option for postmenopausal women. To date, there is no published conceptual framework that integrates computer game psychology with health behavior psychology, although the “games for health” movement emphasizes the recreational nature of games as a key to motivation [63]. Physical activity may be perceived by some adults as more duty than leisure; as unpaid work rather than as temptation. In other words, when it comes to physical activity, kids play, adults “work out”. Video dance and other interactive games might promote adherence to physical activity by aligning delayed and immediate rewards, combining duty with pleasure and leisure, and turning unpaid work into temptation.

### Sample

Participants must be female, age 50–65, not currently exercising at least 20 min, three times per week, and have a BMI of 25 or higher. Exclusions are largely related to medical safety and include history of osteoporosis, osteoporotic fractures, active cardiovascular disease, uncontrolled hypertension, weight bearing pain that would limit exercise, seizure disorder or any medical condition or medication that would limit the safety of the study. The study has been approved by the institutional review board and all subjects sign informed consent.

### Interventions

The three interventions are characterized in the following paragraphs.

Both exercise interventions are designed to offer equivalent contact with staff, access to center exercise resources, similar education about exercise and comparable weaning to independent exercise after 3 months. The main difference between the two exercise arms is the type of exercise (dance versus brisk walking). Delayed entry controls perform baseline and follow up testing for 6 months, and are then offered their preferred choice of video dancing and/or walking.

#### Walking

The overall goal is to increase the duration and speed of walking, using structure and supervision for the first three months, followed by reduced support in the second three months. At the beginning of each walking session, the participant performs a 5 min warm-up consisting of lower extremity stretches. For the first two week initiation phase, each participant must come to the exercise center at least twice a week and walk on the 180-ft oval indoor track for 30 min, alone or in small groups. They are encouraged to gradually increase effort and duration to a target of 150 min per week of brisk walking [64,65], using the track or their own preferred walking location. The number of laps to yield various total distances is posted in the area. Participants are taught to use Borg’s ratings of perceived exertion [66] and self-monitored heart rate to target their level of activity. During the two week initiation phase, a research assistant is present in the walking area, to provide assistance and advice, and to maintain equivalent attention to the dance exercise arm. The research assistant keeps a record of the participant’s Borg ratings, measures pulse and blood pressure weekly, keeps the overall record of session participation, records participant comments, makes observations, and records reasons for missed sessions as reported by the participant. Prior to progression and independent exercise, the research staff member will confirm that the participant has safe blood pressure values with exercise (eg does not drop 20 mmHg or increase to over 160 mmHg with exercise.

After the first two weeks, the next 10 weeks include once weekly supervised sessions and additional sessions either at the center or in preferred community settings. The recommended goal is a minimum of 150 min per week of exercise in sessions of at least 10–15 min duration. Participants may elect to exercise alone or with another participant, and begin to keep their own record of session participation. Participants are given pedometers in order to help them monitor their progress and track their daily steps. After 12 weeks, the participant enters a transition phase for a further 3 months. During this time, the participant may sign up for as many unsupervised walking sessions as they wish at the Center and/or can walk in other settings as preferred.

#### Video dancing

This intervention uses a commercially available product called Dance Dance Revolution (DDR) (Konami). This video-game based dancing system uses a game player, force sensing pad and software. Nothing is attached to the body. This version of interactive video dance games is played while standing on a dance pad of about 3 ft by 3 ft, on which there are four 1 ft squares. On each square there is an arrow pointing either forward, backward, right or left. For the version used here, the pad is connected to a video monitor via a videogame system. The monitor provides direction to the player via a system of scrolling arrows which typically rise from the bottom to the top of the screen. As the arrows scroll, they cross a set of corresponding arrow silhouettes. The dancer must step on the corresponding dance pad arrow as the scrolling arrow crosses its silhouette. The player can also be asked to hop on to two arrows at once. The step sequences are set to a wide range of music and become more complex and frequent as the dancer gains skill. The step frequency and pattern of beginner dances is like marching or walking, but the game gradually introduces more varied rates and irregular patterns, that may challenge fitness, coordination, attention and muscle power. Most single dances last 90 s to two minutes. The game provides feedback in two ways; a report of accuracy concurrent with each single arrow, and after each dance via a “results screen” which provides a “grade” and a summary of step accuracy. As the dancer gains skill, the grade improves and the dancer can “win” new dances.

Participants who are randomized to the dancing arm first receive a full orientation to the structure and navigation of the system and the controls by our trained research staff. During the two week initiation phase, each participant begins with a series of 8 orientation lessons are available in the dance program, while receiving advice and encouragement from a staff member. Participants who have mastered the lessons then select from the Beginner Level of dances and perform at least 4 supervised 30 min sessions over the 2 week initiation phase. During this time, they are taught to navigate the game system on their own. At the beginning of each dance session, the participant performs a 5 min warm-up consisting of walking and lower extremity stretches. By the end of the initiation phase, the participant is expected to demonstrate the ability to navigate the game system and confirm safe blood pressure values with exercise. After the initiation phase, and for the rest of the first 3 months, the participant is expected to attend at least one supervised session per week. Participants may use the center for additional, unsupervised sessions and/or they can take a dance system home. Those who take a system home are instructed in system assembly and trouble shooting. After the first 12 weeks, the dancers transition to a period of independent activity for 12 more weeks.

Both exercise arms receive brief behavioral intervention sessions for safety orientation, exercise education and adherence promotion. Participation in these sessions is separated by treatment arm.

#### Delayed entry control

Participants who are randomized to the delayed entry non-exercise control group receive the American Heart Association pamphlet, but no direct support for exercise implementation. After they have completed six months of follow up, they are invited to select any combination of dancing and walking that they prefer and then receive support and instruction according to the protocols described above.

### Measures

Adherence, the primary outcome, is assessed as minutes per week of moderate or greater physical exercise activity assessed using accelerometers and activity diaries. Endurance is assessed by timed 2 km walk [67] strength by one repetition max (1-RM) knee extension, body composition by Lunar Prodigy DXA scanner for lean body mass and total fat mass, abdominal obesity by waist circumference, vascular health by blood pressure, pulse, lipid levels, fasting glucose, fasting insulin and C reactive protein, and balance by timed one foot stand [68] and timed narrow walk [69]. We also assess demographics (age, ethnicity, education, occupation, living situation, family structure and income group), medical conditions and medications by self reported data on physician diagnosis of medical conditions [70] and current prescribed and over the counter medications, sleep quality by Pittsburgh Sleep Quality Index [71], mood by CES-D [72], menopausal history and current symptoms by the Stages of Reproductive Aging Workshop [73] and the MENOQOL [74], balance confidence by the Activities-specific Balance and Confidence Scale (ABC)) [75], exercise and computer use history by the Historical Leisure Activities Questionnaire (HLAQ) [76]. We modified the HLAQ to include 6 cognitive leisure activities, as well as computer and computer game use. We assess self-efficacy for exercise by the Self-efficacy for exercise scale (SEE) [77], personality by the NEO Personality Inventory [78](REF), and leisure preferences and exercise enjoyment by the Physical Activity Enjoyment Scale [79] and items from a computer game enjoyment inventory [80].

### Cognitive Domains to Be Assessed in the Clinical Trial

We use elements of the Repeatable Battery for the Assessment of Neuropsychological Status (RBANS) to assess two cognitive domains [81].The visuospatial/constructional domain is assessed with two subtests: a) copying a complex figure and b) line orientation (matching lines of varying spatial orientation to a given sample). The attention domain is also assessed by two subtests that require focused concentration and rapid shifting of attention between presented stimuli. To measure attention deployment and visuo-spatial skills, participants also perform the Useful Field of View test (UFOV) [82] and Step Reaction Tasks (SRTs) [83]. The Useful Field of View test is a computer-based measure, where participants are seated in front of a computer screen and must localize a quickly flashed target among a multitude of distractors. The UFOV provides a controlled laboratory paradigm to assess selective and divided attention, assessing deployment of visual attention over space. It has been shown to be a good predictor of driving accident rates in older persons [84]. In the Step Reaction Tasks, participants step in varying directions (front, back, left or right) in response to a visual cue from a computer screen, while wearing thin force-sensors attached to the soles of the shoes. The tests vary in cognitive complexity and the amount of motor planning required for stepping after the visual cue is given. Data is collected as reaction time to the varying visual cues.

FMRI assessment, the neuroimaging element of the trial is performed on a subset of participants who volunteer to participate in this substudy. Participants from all three arms are eligible. Participants are being scanned on a Siemens 3 T Allegra scanner with the acquisition of high-resolution anatomical images, diffusion-weighted imaging for the assessment of white matter integrity, a resting state scan to determine resting state functional connectivity, and two task-evoked fMRI paradigms. One of the task-evoked paradigms, the DSST has been described in detail above. In addition to the DSST, a task-switching paradigm is also being implemented. The task-switching paradigm requires the participant to respond to whether letters presented on a computer display are in an uppercase or lowercase font or to identify whether the letter is a consonant or a vowel. In this task-switching paradigm the participant is provided with a cue instructing them as to which task to perform in the upcoming trial. When the participant performs the same task sequentially (e.g. uppercase/lowercase judgment followed by another uppercase/lowercase judgment), the condition is considered a ‘repeat’ trial. However, when the participant performs one task that is followed by the alternative task (e.g. uppercase/lowercase judgment followed by a consonant/vowel judgment) the condition is considered a ‘switch’ trial. Switch trials take longer to perform than repeat trials, a difference in response times often referred to as a ‘switch cost’. Older adults often show larger switch costs than younger adults, yet some aerobic exercise interventions have found that training can improve task-switch performance and reduce switch costs [45]. Task-switching is often considered an exemplar of an executive functioning task and is supported by prefrontal and parietal brain circuits [28]. The benefit of using the task-switching paradigm in this study is that it requires cognitive skills that are commonly used in video game performance including executive control and psychomotor control. In addition, switching between responses, demands, decisions, and tasks is an inherent component of most video games and expert video gamers outperform their more novice peers on task-switching tasks [25]. Hence, we reasoned that task-switching might not only be amenable to a dancing intervention, but the brain networks involved in supporting task-switching performance might be the most affected by the training intervention.

### Analyses

Randomization is based on a computer based random number generator which creates assignments that are numbered sequentially and placed in sealed envelopes. Participants are assigned to a treatment arm by opening the next numbered envelope after they have completed the consent and baseline assessment process. The study sample size was determined based on achieving 80% power to detect a 20 min difference in minutes per week of physical activity at 6 months between dancers and walkers, with the target recruited sample increased by 15% to account for potential dropouts. To determine whether video dance induces greater changes in health indicators, compared to controls, we will compare change from baseline to post intervention between groups. To examine whether video game dance activity is superior to brisk walking in promoting balance, attention and visual-spatial skills, we will compare baseline to post intervention changes between dance and brisk walking groups. To assess factors that influence activity preference, we will compare final scores on the Physical Activity Enjoyment Scale between dancers and walkers and compare minutes dancing versus walking among delayed entry controls during their phase of free choice activity.

## Conclusion

This randomized clinical trial may provide new information about the cognitive effects of interactive videodance. It is also the first trial to examine physical and cognitive effects in older women. Interactive video games may offer novel strategies to promote physical activity and health across the life span.

## Competing Interests

Jovancevic: none. Rosano: none. Perera: none. Erickson: none. Studenski: Dr. Studenski previously received grant support from Humana Inc. for studies related to aging and videodance.

## Authors’ contributions

JJ planned the tests of visual attention and step reaction times and drafted the manuscript. CR and KE planned the neuroimaging approach and collected and analyzed the preliminary imaging data., SP planned the statistical analyses and advised on study design and power. SS designed the trial, collected preliminary data and drafted the manuscript. All authors reviewed and approved the final manuscript. .

## Pre-publication history

The pre-publication history for this paper can be accessed here:

http://www.biomedcentral.com/1471-2318/12/23/prepub
